# BK virus nephropathy in a heart transplant recipient

**DOI:** 10.1590/2175-8239-JBN-2020-0049

**Published:** 2021-01-22

**Authors:** John Fredy Nieto-Ríos, Diego Armando Benavides-Henao, Arbey Aristizabal-Alzate, Carol Morales-Contreras, Diana Carolina Chacón-Jaimes, Gustavo Zuluaga-Valencia, Lina María Serna-Higuita

**Affiliations:** 1 Hospital Pablo Tobón Uribe, Department of Nephrology and Kidney Transplant, Medellín, Colombia.; 2 University of Antioquia, Nephrology Section, Department of Internal Medicine, Medellin, Colombia.; 3 Eberhard Karls University, Institute for Clinical Epidemiology and Applied Biometrics, Tuebingen, Germany.

**Keywords:** Polyomavirus, BK virus, BK Virus Nephropathy, Organ Transplantation, Heart Transplantation, Immunosuppression., Poliomavírus, Vírus BK, Nefropatias, Transplante de Órgãos, Transplante de Coração, Imunossupressão.

## Abstract

BK virus nephropathy in kidney transplantation is widely recognized as an important cause of graft dysfunction and loss. In the case of transplants of organs other than kidney, BK virus nephropathy in native kidneys has been recognized as a cause of chronic kidney disease, which is related with immunosuppression; however, the diagnosis is usually late because the renal dysfunction is attributed to other causes, such as toxicity by anticalcineurinic drugs, interstitial nephritis due to medications, hemodynamic changes, diabetes, hypertension, etc. We report a case of BK virus nephropathy in a patient who underwent heart transplantation due to peripartum cardiomyopathy. The kidney biopsy reported active chronic tubulointerstitial nephritis associated with late stage polyomavirus nephritis and the blood viral load for BK virus was positive (logarithm 4.5). The immunosuppressive treatment was reduced, and after two years of follow-up, the patient had stable renal function with a serum creatinine of 2.5 mg/dL (GFR of 23.4 mL/min/1.73m2). We recommend that the BK virus be considered as a cause of renal dysfunction in heart transplant recipients, with the aim of detecting its replication in time to reduce immunosuppressive therapy before irreversible compromise of renal function may manifest.

## Resumo

A nefropatia pelo vírus BK no transplante renal é amplamente reconhecida como uma importante causa de disfunção e perda do enxerto. No caso de transplantes de órgãos que não sejam rins, a nefropatia pelo vírus BK em rins nativos tem sido reconhecida como uma causa de doença renal crônica, que está relacionada com imunossupressão; entretanto, o diagnóstico é geralmente tardio porque a disfunção renal é atribuída a outras causas, tais como toxicidade por drogas anticalcineurínicas, nefrite intersticial devido a medicamentos, alterações hemodinâmicas, diabetes, hipertensão, etc. Relatamos um caso de nefropatia pelo vírus BK em um paciente que foi submetido a transplante cardíaco devido à cardiomiopatia periparto. A biópsia renal relatou nefrite túbulo-intersticial crônica ativa associada à nefrite por poliomavírus em estágio avançado e a carga viral sanguínea para o vírus BK foi positiva (logaritmo 4,5). O tratamento imunossupressor foi reduzido, e após dois anos de acompanhamento, o paciente apresentava função renal estável com creatinina sérica de 2,5 mg/dL (TFG de 23,4 mL/min/1,73m2). Recomendamos que o vírus BK seja considerado como uma causa de disfunção renal em receptores de transplante cardíaco, com o objetivo de detectar sua replicação a tempo de reduzir a terapia imunossupressora antes que um comprometimento irreversível da função renal possa se manifestar.

## Introduction

BK viruses are circular, double-stranded DNA viruses^1^^-^ 4 that typically cause asymptomatic infections in the pediatric population, persisting latent in the renal epithelium and lymphocytes with minimal episomal replication^2^^,^ 5 ^,^ 6. In immunosuppressed patients, this virus is associated with different complications, including BK virus nephropathy, ureteral stenosis, and hemorrhagic cystitis^1^^,^ 3 ^,^ 7.

At present, the exact route of transmission is not known, but it is believed that the virus is usually acquired via the air in childhood^8^. It is postulated that the BK virus enters the blood by infecting mononuclear cells that circulate through the tonsil tissue, allowing it to spread to distant sites, including the kidneys, spleen, thyroid, and pancreas^5^. Latent infection typically compromises the genitourinary tract, where it establishes a latency for life. Reactivation of the virus can occur in immunosuppressed patients, particularly in patients receiving immunosuppressive therapies^6^^,^ 9. BK virus nephropathy is recognized as an emerging problem in renal transplant recipients; approximately 30-50% of renal transplant patients present BK viruria one month after renal transplantation^10^ and 5-10% develop BK virus nephropathy, of which 50-80% develop renal graft failure^9^^,^ 11. The risk factors for the development of BK virus nephropathy include age over 50, male sex, rejection treatment, prolonged cold ischemia times, lymphocyte-depleting induction, and the use of immunosuppressants such as tacrolimus/mycophenolate^12^.

In recent years, cases of BK virus nephropathy have been reported in native kidneys of patients who have undergone bone marrow transplantation, as well as in transplantation of other non-renal solid organs, such as heart, liver, and lung^10^^,^ 13. We present here the case of a heart transplant patient who developed a BK virus nephropathy, with a late diagnosis of this complication resulting in advanced chronic kidney disease.

## Clinical case

A 39-year-old woman with a diagnosis of peripartum cardiomyopathy who received a heart transplant in October 2014. She received induction with Basiliximab and methylprednisolone. In addition, she was given a maintenance treatment with extended-release tacrolimus XL, 7 mg daily, everolimus 1, twice daily, and prednisolone, 5 mg/day. She had two acute rejection episodes during the first year post-transplant, and was managed with pulse methylprednisolone, with good results. There was no history of kidney disease and her renal function was stable, with creatinine of 0.88 mg/dL and glomerular filtration rate (GFR) of 102 mL/min/1.73m^2^ during the first year post-transplant. Management was exclusively performed by the cardiac transplant group and routine monitoring of polyomavirus with viral load BK or urine cytology was not done. In 2016, she presented an elevation of serum creatinine up to 1.9 mg/dL, with a GFR of 32.6 mL/min/1.73m^2^. At that time, tacrolimus trough level was 7.2 ng/mL and everolimus, 5.2 ng/mL. Toxicity by anticalcineurinics was suspected; therefore, tacrolimus was reduced to 4 mg daily and creatinine value returned near to the baseline value (creatinine 1.25 mg/dL, GFR 54.1 mL/min/1.73m^2)^; no kidney biopsy was performed. In March 2017, creatinine raised to 2.69 mg/dL, with a TFG of 21.4 mL/min/1.73m^2^, for which she was hospitalized. The patient stated she did not present any symptom. At physical examination, she was observed in good general condition, heart rate of 80 beats per minute, blood pressure of 130/90 mmHg, respiratory rate of 15 per minute, afebrile. Additional studies were conducted: ultrasound of the renal tract showed normal renal size, but increased echogenicity; urinary microscopy and culture analyses were negative, with no hematuria, pyuria or casts; echocardiogram with adequate cardiac function; serological tests for HIV, syphilis, hepatitis virus B and C were negative; tacrolimus trough level of 5.2 ng/mL, and everolimus of 5.98 ng/mL (Table 1). Management was initiated with intravenous hydration, and the dose of tacrolimus XL was decreased to 2 mg daily, but there was no improvement of kidney function; a kidney biopsy was planned.

**Table 1:** Laboratory tests results

Urine Tests	
Density	1.005
Proteinuria	Negative
Glycosuria	Negative
Leukocytes	0-5
Erythrocytes	0-2
Bacteria	Scarce
Test for infectious diseases	
Bk virus, viral load (blood)	33800 copies/mL
AgS Hepatitis B	Negative
Antibodies for hepatitis C	Negative
Antibodies for human immunodeficiency virus	Negative
Immunological Tests	
Serological test for syphilis	Negative
Antinuclear antibodies (ANAS)	Negative
Antibodies anti-DNA	Negative
Complement C3 y C4	Normal
Blood Tests	
Sodium	140 mmol/L
Chloride	108 mmol/L
Potassium	3.68 mmol/L
Calcium	8.5 mg/dL
Phosphorus	3.3 mg/dL
Lactate dehydrogenase	188 U/L
Parathormone	81 pg/mL
CPK total	126 U/L
Albumin	4 g/dL
Hemoglobin	11.3 g/dL
Hematocrit	26.80%
Leukocytes	4900 mm^3^
Platelets	168000 mm^3^
Neutrophils	66%
Lymphocytes	20%
Echocardiography	
Left ventricular ejection fraction: 60%	

AgS: surface antigen.

**Figure 1:**
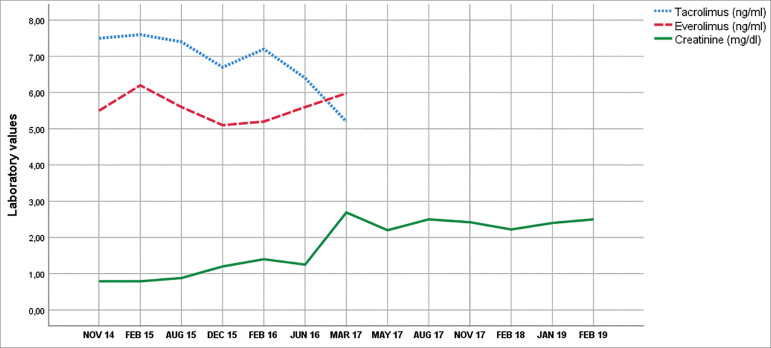
Laboratory values during follow up.

**Figure 2:**
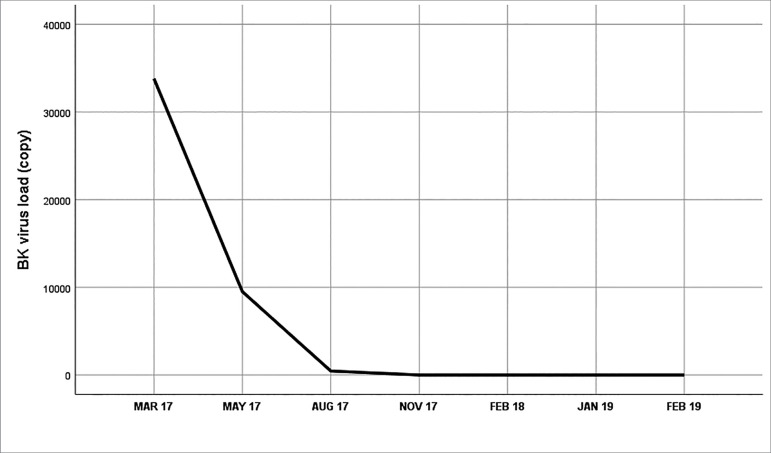
BK viral load (number of copies).

**Figure 3:**
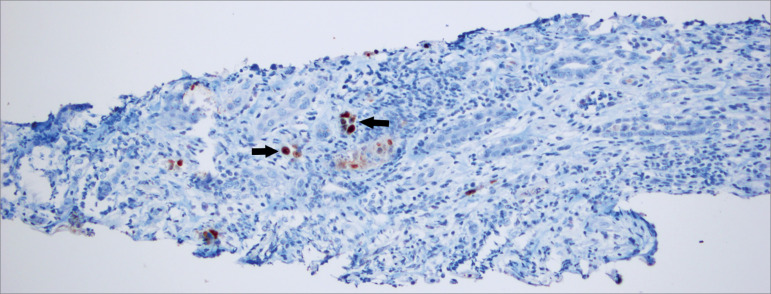
Renal biopsy report: active chronic tubulointerstitial nephritis, with positive immunohistochemistry for SV40, consistent with stage C polyomavirus nephritis (arrows). Image courtesy of Department of Pathology, Fundación Santa Fe de Bogotá, Colombia.

The kidney biopsy revealed active chronic tubulointerstitial nephritis, associated with late stage polyomavirus nephritis (Figure 1). PCR for BK virus was performed and the result was positive at 33800 copies/mL in blood (logarithm 4.5). Tacrolimus was withdrawn; creatinine levels stabilized between 2.2 and 2.4 mg/dL, without further elevation in post-discharge controls. Her viral load started to decline until reaching undetectable values. Patient progress is summarized in Figures 2 and 3. The patient did not presented episodes of cardiac rejection at 3 years of follow-up; the last creatinine measure was 2.5 mg/dL, corresponding to a GFR of 23.4 mL/min/1.73m^2^.

## Discussion

Polyomavirus nephropathy is a severe opportunistic infection that occurs in kidney transplant patients. In rare cases, it also affects the native kidneys of transplant recipients of other organs^14^, and it can lead to terminal chronic kidney disease of the native kidneys^8^. In recent years, the reporting of BK virus infection has increased in transplant recipients of heart, lung, liver, pancreas, and kidney plus pancreas^8^. In addition, there are reports of BK virus infection in the urinary system of bone marrow transplant recipients, in whom it manifests primarily with hemorrhagic cystitis^8^^,^ 15.

In the context of cardiac transplantation, BK virus infection of the urinary system has been reported mainly during rejection episodes, associated with an increase in immunosuppressant drugs. In some studies, BK viruria of up to 19% has been reported in heart transplant recipients, and viremia up to 5%, but BK virus nephropathy is unusual in this population. It has been suggested that additional damage to the native kidneys is required for the development of BK viral nephropathy^8,16^. Vigil D *et al*. reviewed the literature on heart transplants with BK virus infection. Eleven patients were reported, nine males, 81% of cases associated with rejection, of which 72% had terminal chronic kidney disease, with a mortality of 27%^8^. In the case reported here, the patient was a heart transplant recipient, with a previous episode of acute rejection, so she was strongly immunosuppressed. In addition, she presented a progressive increase in creatinine, initially attributed to toxicity by anticalcineurinics, but due to the poor response to the initial therapy, a renal biopsy was performed, and viral load was requested for BK virus, which was positive. These findings allowed the diagnosis of BK virus nephropathy, and due to the late diagnosis, the patient developed stage 4 chronic kidney disease^12^^,^ 17 ^,^ 18.

Detection of tubular or urothelial cells with inclusions of BK virus cells in urine is a useful tool for the diagnosis of this infection in the urinary system. These cells are known as Decoy cells for their similarity with tumor cells in Pap smears. Decoy cells have a sensitivity of 25% and a specificity of 84%^12^ but this study was not performed on the patient because initially this diagnosis was not considered. Electron microscopy in urine samples has a sensitivity and specificity of 100% but is not available in many centers^12^. Viral detection by PCR is a useful tool, widely available and with high sensitivity (100%) and specificity (78%) for diagnosis (PCR of the BK virus in urine: sensitivity 100%, specificity of 78%; PCR of the BK virus in blood: sensitivity of 100% and specificity 88%). It is also a useful prognostic parameter, since high levels of viruria or viremia correlate with the presence of BK virus nephropathy^12^.

Kidney biopsy is considered the "gold" diagnostic standard. It is usually indicated when viremia is greater than 10,000 copies/mL, with or without creatinine elevation, and with kidney dysfunction without recognizable cause^12^. In addition, immunohistochemistry for SV40 T antigen must be performed, which, when positive, allows to detect with high accuracy the viral infection. However, it should be taken into account that sometimes the infection is focal, so an insufficient renal biopsy may not detect the infection^8^^,^ 18 ^-^ 20. The original Banff classification recognizes three histological patterns: a first early stage without tubular cell necrosis (stage A); a second stage of active nephropathy with tubular cell necrosis (stage B); and a late third stage characterized by advanced fibrosis (stage C)^21^. This classification correlates with the risk of CKD progression, as stage A is an early stage without fibrosis and completely reversible, while stage C is usually irreversible, as in this patient's case.

Currently, there is no standard therapy for BK virus nephropathy^8^. Certain drugs have demonstrated antiviral properties *in vitro* (quinolones, leflunomide, cidofovir, statins), but they have not yet shown significant results in clinical studies^12^. Intravenous immunoglobulin, combined with the reduction of immunosuppressive therapy, may have some initial beneficial effect in the clearance of viremia, but it is followed by an increase in viremia and BK virus nephropathy^22^. In our patient, immunoglobulin was not prescribed, as the use of immunoglobulin in BK viral nephropathy is not currently approved in Colombia. The current therapeutic approach for BK virus infection consists in the reduction of immunosuppression or substitution of the different immunosuppressive pharmacological groups. Since the efficacy of BK viral nephropathy treatments is limited, conducting periodic screening tests in the period after transplantation or during rejection therapy are recommended to prevent this infection. In renal transplantation, the measurement of BK virus viral load in serum is recommended monthly from one to twelve months, and then every three months^19^. In other types of transplants, screening frequency has not yet been established. However, the systematic screening of viremia and BK viruria within post-transplant follow-up, especially in patients at high risk for this infection, may allow timely detection of the virus and an early modification of the immunosuppressive scheme to avoid chronic kidney damage^12^^,^ 17 ^,^ 23. In the case reported here, the treatment aimed to decrease immunosuppression by suspending the anticalcineurinic agent, which allowed to control the infection without rejection. The patient was already receiving a mammalian target of rapamycin inhibitor (MTOR) (everolimus), which was maintained to avoid rejection and because this drug has been attributed with antiviral properties, although in the case of BK virus ifection, its effectiveness is controversial^18^.

In summary, BK virus nephropathy in transplant patients other than kidney transplant recipients is a silent entity that can lead to chronic kidney disease with increased morbidity and mortality. We therefore propose to consider the BK virus infection as a cause of renal dysfunction in heart transplant recipients, with the aim of detecting its replication in time to reduce immunosuppressive therapy before irreversible impairment of renal function may manifest.
